# Tracking Lead in Environmental Media in the City of Onitsha, Southeast Nigeria

**DOI:** 10.5696/2156-9614-9.24.191202

**Published:** 2019-11-27

**Authors:** Timothy Iyobosa Asowata, Akinade Shadrach Olatunji

**Affiliations:** 1 Department of Applied Geology, Federal University of Technology, Akure, Nigeria; 2 Department of Geology, University of Ibadan, Ibadan, Nigeria

**Keywords:** lead isotope, anthropogenic sources, environmental media, city of Onitsha

## Abstract

**Background.:**

The enrichment of lead (Pb) in a rapidly expanding urban environment is largely caused by industrial and anthropogenic activities. However, very few studies have come from sub-Sahara Africa as a whole, in spite of the increased rate of population, industrialization and urbanization in this region. The city of Onitsha is the commercial heartland of southeast Nigeria.

**Objectives.:**

To determine the concentration of Pb in soils and sediments in Onitsha and the surrounding area and to identify the possible sources of Pb content in the environmental media.

**Methods.:**

One hundred and sixty-two (162) samples (120 top and subsoils, and 42 stream and side drain sediments) were collected from the city of Onitsha, Nigeria to determine Pb concentrations, identify the main sources of Pb in this region, and determine its fate in soil and sediments using Pb isotopes. Thirty (30) 15 g clay fractions of soil and sediment samples, and a sample each of galena (from the Lower Benue Trough), coal and soot from vehicle exhaust and battery cells were collected from the city and analyzed for lead isotopes (Pb204, Pb206, Pb207 and Pb208) using ultra-trace inductively coupled plasma emission spectrometry.

**Results.:**

The distribution and concentration of Pb in the soil of Onitsha was observed to be influenced by land use patterns with very elevated concentrations of Pb observed for mechanic and metal workshop samples (1444.3, 1067.5, 1048.1, 1730.5 and 580.5 ppm); active waste dump samples (448.4 and 311.9 ppm); and farmland and garden samples (366.2 ppm). The concentration of Pb in the sediments also showed varying elevated concentrations across locations, ranging from 45.7–540.1 ppm. A comparison with the control samples revealed that the Pb concentrations measured in the environmental media were several folds higher than that of the control. The Pb isotope analysis indicated that most of the Pb in environmental media was anthropogenic in origin and had been predominantly contributed by unsustainable environmental practices such as indiscriminate waste dumps, hydrocarbonbased products emissions, by-products from mechanical workshops that have been haphazardly constructed in the city, and industrial plants located within urban areas.

**Conclusions.:**

The relatively higher concentrations of Pb in soils and sediments were found to be influenced by land use, as also observed in the Pb isotope readings, which will, over time, adversely affect environmental media and biota.

**Competing Interests.:**

The authors declare no competing financial interests.

## Introduction

The growth of most Nigerian cities in the last 3 decades has been phenomenal and often poorly controlled. The cities have experienced unplanned urbanization and industrialization, which has heavily impacted the quality of environmental media.^1–5^ Onitsha is a major city in southeastern Nigeria with thriving commercial and industrial activities alongside a large human population. The city had been reportedly described as one of the most polluted cities in the world due to impaired air quality.^6^

A recurring pollutant of environmental media in Nigeria is lead (Pb). Lead has been documented to be a pollutant in most previous studies undertaken in Nigerian cities.^6^ This is particularly true around routes with large traffic volume, industrial areas and commercial centers.

Lead has had many uses throughout human history. Despite its toxicity, Pb continues to find applications in several manufacturing activities.^7^,^8^,^9^ Lead has been phased out from use as an anti-knocking agent in gasoline worldwide after being found to be a major source of Pb pollution in the environment.^9^,^10^ However, Pb has many useful applications in the chemical and petrochemical industries.^10^

Several studies have documented the legacy of Pb pollution in the environment in developed countries, but very few have been conducted in the developing nations of Africa. One useful method for delineating the sources of Pb pollution is the application of the Pb isotope ratio of ^206^Pb/^207^Pb, especially as it relates to traffic emissions.^11–14^ One study used the ^206^Pb/^207^Pb ratio to determine that a significant amount of Pb (70%) concentration in plants in northern Sweden originated from anthropogenic sources, due to the differences in Pb isotopic ratio between natural and anthropogenic sources.^14–15^

Lee *et al.* also used this isotope ratio to determine that Pb in the road sediments of Seoul, Korea was mainly derived from industrial sources rather than from leaded gasoline.^12^ The use of Pb isotopes is a good tool for discriminating various sources of pollution that could be responsible for the deterioration of the quality of environmental media in cities such as Onitsha that have many potential sources of pollution. This is crucial for the development of appropriate pollution mitigation processes. The objectives of the present study were to determine the concentrations of Pb in soil and sediment samples within the city of Onitsha and to identify the possible source(s) of Pb content using Pb isotopes.

## Methods

**Figure 1:**
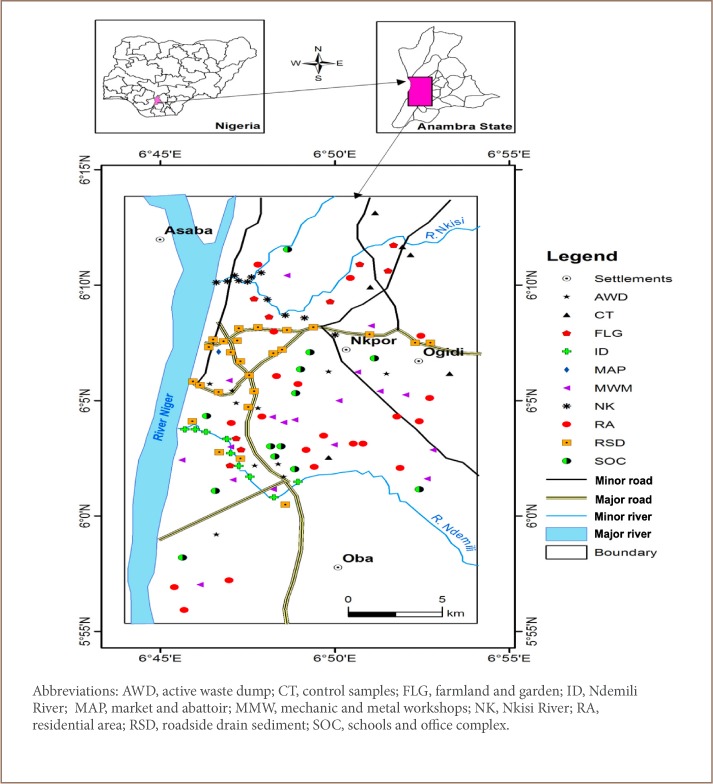
Map of study area^17^

The city of Onitsha is located within latitudes 6°5′ to 6°11′ N and longitudes 6°45′ to 6°53′ E *(Figure 1)*, with an area of about 72 km^2^. The city is characterized by an extensive flood plain with average elevation of 26 m above sea level.

The climate is tropical equatorial, with defined wet and dry seasons. The wet season runs from March through October with a lull in August called ‘August break’, which nearly divides the wet season into two. The remaining months form the city's dry season. Like a good portion of West Africa, the city experiences the Harmattan (dry) season between the months of November and February. The area falls within the tropical rain forest belt with a mean precipitation of 2100 mm.^16^

**Abbreviations**
*EC*: Electrical conductivity
*TDS*: Total dissolved solids

The study area is drained by three rivers, with the river Niger being the main river that runs from the north to the south of the city. The Nkisi and Ndemili Rivers are important tributaries of the river Niger. The Ndemili River in the southern part of the city drains the city from east to west, while the Nkisi River also drains the city from east to west in the northern part of the city *(Figure 1)*. The floodplains of the rivers permit dry-season farming, fishing and sand dredging in the area.

**Figure 2:**
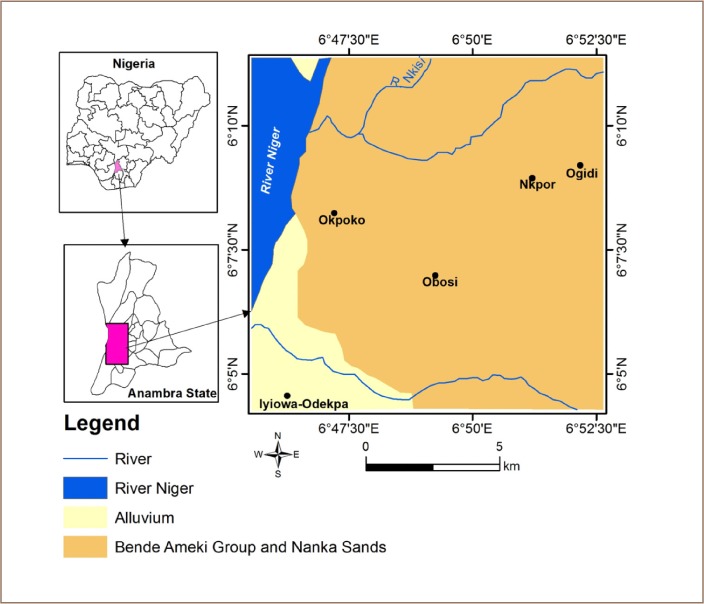
Geologic map of Onitsha area (Sheet 71)^23^

**Figure 3:**
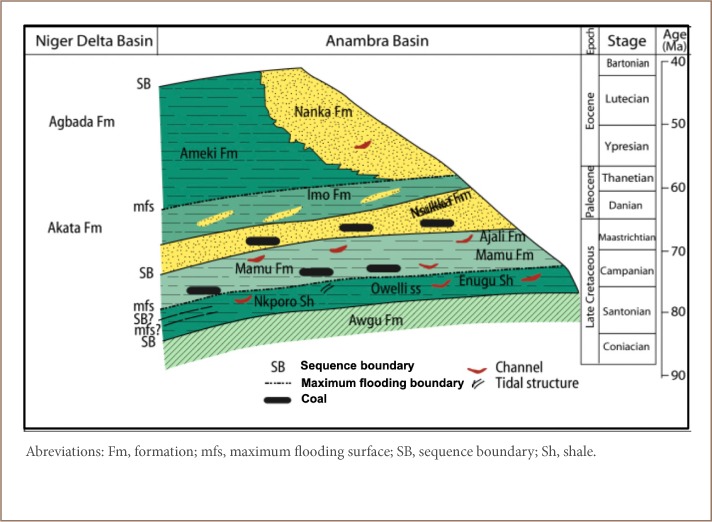
Stratigraphic section of the Anambra Basin from the late Eocene and time equivalent formations in the Niger Delta^22^

The Onitsha area is underlain by the Ameki Formation, although the western edge of the region, bounded by the river Niger, houses alluvium deposits *(Figure 2)*. The Ameki Formation dates from the Eocene and its lateral facies equivalence is the Nanka Formation *(Figure 3)*. The Ameki Formation consists of a series of highly fossiliferous grayish-green sandy clay with calcareous concretions and white clay sandstone. It is comprised of two lithological groups. The lower group is fine to coarse grained sandstone with intercalation of calcareous shale and thin shaly limestone, while the upper group is coarse grained cross bedded sandstone with bands of fine grey-green sands and sandy clay.^18^ Studies have reported that the Ameki Formation has between 364 m - 455 m regressive facies and shallow marine environments.^19–21^ The formation overlies the Imo Formation. Its lateral equivalence is the Nanka sand.^22^

### Sampling

A total of one hundred and sixty-two (162) samples were collected for analysis. These include 85 topsoil (0–30 cm depth), 35 subsoil (30–100 cm depth), 20 stream sediment (11 from the Ndemili River and 9 from the Nkisi River) at an interval of 250 m, and 21 roadside drain sediment samples collected on the drainages of major roads in the study area at an interval of 250 m.^24^ The soil samples were collected using the following land use patterns: residential area; farmland and garden; active waste dump; mechanic and metal workshops; market and abattoir; schools and office complex; and control samples (N = 6, collected from the outskirts of the city in unurbanized areas). Sediments samples were collected from roadside drain sediment; the Ndemili River; and the Nkisi River.

The soil samples were collected with a 1.5 m stainless-steel sampling auger, which enabled researchers to maintain the required depth of 1 m. The sediment samples were collected with the aid of a clean plastic bowl. Three to four subsamples were collected and composited as one representative sample for every location for the soils and sediment samples. This was adopted in order to reduce point source contamination errors.^25–27^

The soil and roadside drain sediment samples were subsequently air dried at room temperature for 72 hours and stored. The stream sediments, collected in plastic bottles, were allowed to settle, the water in the plastic bottle was decanted and the sediment in the clay fraction was allowed to dry at room temperature for 5 to 6 days, and then stored for further preparation. All samples were further sieved to less than 75 μm for subsequent analysis.

Four more samples including one each of galena (from the Pb-zinc (Zn) belt of the Lower Benue Trough), coal (from the Anambra Basin), soot from vehicle exhaust, and soot from used battery cells within the study area were obtained for Pb isotope analysis, in order to provide a basis of comparison of Pb contents in the media and to be able to discriminate the possible sources of Pb found in the environmental media.^27–29^

Fifty (50) g of air-dried soil and sediment samples were measured and transferred into a 200 ml beaker, and 100 ml of distilled water was gradually added and left for 30 minutes to facilitate water movement through the samples. The samples were later stirred occasionally with a glass rod and allowed to stand for 24 hours. An Hach Eco 40 multi and Milwaukee Electric Tool Corporation handheld digital meter were used to measure pH, electrical conductivity (EC) and total dissolved solid (TDS), after they were standardized with appropriate buffer solutions at the Department of Geology, University of Ibadan, Ibadan. This procedure followed previously published works.^29^,^30^

### Geochemical analyses

Prior to geochemical analysis, 0.5 g of soil and sediment samples (N = 162) were weighed into the digestion flask and extracted with modified aquaregia (1:1:1) nitric acid: hydrochloric acid: water for two hours at a temperature of 95°C. The digested samples were then allowed to cool and filtered. The digests were subjected to elemental analysis using ultra-trace inductively coupled plasma emission spectrometry (code AQ250, ACME Laboratories, Vancouver, Canada). The minimum limit of detection for Pb was 0.01 ppm *(Table 1)*. Quality control and assurance were guaranteed by the analyses of various certified reference materials and the analysis of duplicate and blank samples. The results are presented in Table 1.

**Table 1:** Quality Control Results for Lead Content and Isotope Analyses for Soils, Sediments and Reference Materials

**Soils**	**PPM**	**Sediments**	**PPM**	**Reference materials Pb isotope**	**PPM**
SOC 8^a^	45.2	NK 1	94	STD DS9	135.4
SOC 8^a^	45.3	NK 1	94	STD OREAS45EA	12.8
AWD ^b^	20.4	RSD 3	102	STD DS9	130.0
AWD ^b^	20.1	RSD 3	101	STD OREAS45EA	12.4
MMW 4^b^	410.1	**Reference Materials**		STD DS9	121.7
FLG 7^a^	28.0	STD DS9	124	STD OREAS45EA	13.7
FLG 7^a^	29.5	STD OREAS45EA	13	STD DS9	134.2
MAP 1	NA	STD DS9	122	STD NIST-981-1Y	-
MAP 1	NA	STD OREAS45EA	13	STD NIST-983-1Y	
		BLK	<3	BLK	<0.1
		BLK	<3	BLK	<0.1

Minimum limit of detection	0.01		0.01	BLK	0.1

Abbreviations: AWD, active waste dump; BLK, blank samples; FLG, farmland and garden; MAP, market and abattoir; MMW, mechanic and metal workshops; NK, Nkisi River; RSD, roadside drain sediment; SOC, schools and office complex; STD, standard samples

^a^top soil

^b^sub soil

For the Pb isotopes analysis, 15 g each of the silty-clay fractions of soils and sediments (N = 30) as well as a sample each of galena, coal, soot from vehicle exhaust and soot from battery cells were dissolved in aqua regia. Triplicate digestions of each sample were undertaken to improve the level of dissolution and precision of the obtained results *(Table 1)*. Lead isotopes (Pb^204^, Pb^206^, Pb^207^ and Pb^208^) were then analyzed, using ultra-trace inductively coupled plasma emission spectrometry at the ACME laboratory using the appropriate protocol. The results showed no significant deviation (maximum 5.4 - 0.4) in variation from each other, suggesting high precision *(Table 1)*. This same low deviation ± 0.1 was recorded in the analyzed blank samples.

## Results

A summary of the physico-chemical properties of the soils and sediments in the present study (pH, EC and TDS) is presented in Table 2. The pH, EC and TDS ranged from 4.8–7.7 and 6.1–7.3; 6.41–694 μS/cm and 31.6–1059.0 μS/cm; and 4.05–508.00 mg/l and 20.20–668.00 mg/l for the soils and sediments, respectively *(Table 2)*.

**Table 2:** Summary of the Physico-Chemical Parameters of the Soils and Sediments of the City of Onitsha (N = 162)

**Parameters**	**Sediments**		**Soils**	

	Minimum	Maximum	Minimum	Maximum
**pH**	6.10	7.30	4.8	7.70
**EC** (μS/cm)	31.60	1059.00	6.41	694.00
**TDS** (mg/l)	20.20	668.00	4.05	508.00

Abbreviations: EC, electrical conductivity, TDS, total dissolved solids

Generally, it was observed that pH was influenced by land use activities. While the soils collected from farmland and gardens recorded slightly acidic pH, the soils collected from the mechanic and metal workshops exhibited slightly alkaline pH. The low pH observed for garden and farmlands soil may be connected with the decaying organic matter present combined with the intense rainfall conditions in the area.

Roadside drain sediments had higher EC and TDS compared to the soils in the study samples. This may be a result of the accumulation of materials by the roadside drain sediments and longer interaction of these materials with sediments compared to soils where materials may be more easily removed by flowing water or wind.

The Pb content in both soils and sediments varied from location to location (*Table 3*). It was observed that the Pb content concentration in the vicinity of the mechanic and metal workshops and active waste dumps were found to be higher relative to other land use soils. Similarly, mean Pb content concentrations were relatively higher in roadside drain sediment compared to the mean content of Pb in the Nkisi and Ndemili river sediments (*Table 3*).

**Table 3:** Summary of Pb Concentration (ppm) in Soils and Sediments from the Study Area (N = 162)

**Land Use**	**Minimum**	**Maximum**	**Mean**
Residential area^a^	12.50	257.30	68.07
School and office complex^a^	6.40	253.10	61.01
Farmland and gardens^a^	10.90	366.20	51.44
Market and abattoirs^a^	16.30	270.20	111.9
Active waste dump^a^	20.40	448.40	149.1
Mechanic and metal workshop^a^	22.40	2026.40	379.01
Ndemili River sediment^b^	19.70	180.30	106.95
Nkisi River sediment^b^	24.10	83.70	50.00
Roadside drain sediment^b^	45.70	540.10	151.37
Control	7.20	28.10	18.02

^a^ Soil samples categorized by land-use

^b^ Sediment samples

Abbreviation: STD; standard deviation

The Pb content concentration results for topsoil and subsoil showed varying concentrations *(Table 4)*. The mean Pb content concentration in topsoil was higher compared to subsoil, suggesting that the topsoil received a higher load of Pb content in the study area relative to the subsoil, which indicates that the Pb content in the geo-media may be of anthropogenic rather than geogenic origin.

**Table 4:** Summary of Lead Content in Topsoil and Subsoils in the Study Area (ppm)

	**Topsoil** (0 – 30 cm)				**Subsoil** (>30 – 100 cm)			

**Element**	Minimum	Maximum	Mean	Standard deviation	Minimum	Maximum	Mean	Standard deviation
Pb	7.02	1730.50	167.99	292.80	6.40	2026.40	98.66	335.72

Abbreviation: Pb, lead

## Discussion

The mean concentrations of Pb in soils of various urban cities around the world as well as those in the present study are presented in Table 5. Higher soil Pb concentration were found in the study area (Onitsha) compared with most other cities.

**Table 5:** Global Comparison of the Mean Concentration of Lead in Soils and Sediments of Urban Areas with the Study Area (ppm)

**City**	**Soils Pb**	**City**	**Sediments Pb**
Idrija, Slovenia^30^	49.40	Seoul City, South Korea^9^	214.30
Berlin, Germany^28^	76.60	Uijeongbu City, South Korea^27^	534.00
Ibadan, Nigeria^3^	95.10	Birmingham, England^32^	48.00
Norway^11^	52.00	Lagos Lagoon, Nigeria^2^	20.27
Murcia, Spain^29^	21.90	Kottul, India^31^	43.46
Benin City, Nigeria^1^	232.31	Aqaba City, Jordan^36^	206.00
Kowloo, Hong Kong^31^	94.60	Amman, Jordan^35^	270.50
		Yarlung Tsangpo, Tibet^37^	536.00
**Present study**		**Present study**	
Onitsha, Nigeria	167.99	Ndemili River	106.95
		Nkisi River	50.00
		Roadside drain sediment^*^	151.37

Similarly, the mean Pb concentration in the sediments of the study area is similar to most of the cities referenced for comparison, with the exception of sediments from Seoul and Uijeongbu City, South Korea, and Amman, Jordan and the Yarlung Tsangpo River, Tibet, where mean Pb concentrations were significantly higher than the study area sediments.

Variation was found in the distribution and concentration of Pb in the environmental media of Onitsha *(Table 3)*. For soils, Pb distribution was observed to be influenced by land use patterns with very elevated concentrations of Pb observed for mechanic and metal workshop samples (1444.3, 1067.5, 1048.1, 1730.5 and 580.5 ppm); active waste dump samples (448.4 and 311.9 ppm); and farmland and garden samples (366.2 ppm) *(Table 3)*. A comparison of the obtained results with the control samples (15.7, 7.2, 22.1, 18.6, and 16.1) showed that Pb concentrations ranged from 12- to 20-fold higher than for the control samples. These results indicate that the soils of Onitsha have received significant Pb inputs from various anthropogenic sources.

Furthermore, topsoil was generally found to have higher Pb concentrations than subsoil *(Table 4)*, a further indication that the sources of the Pb in the soils have a recent anthropogenic source.

**Figure 4:**
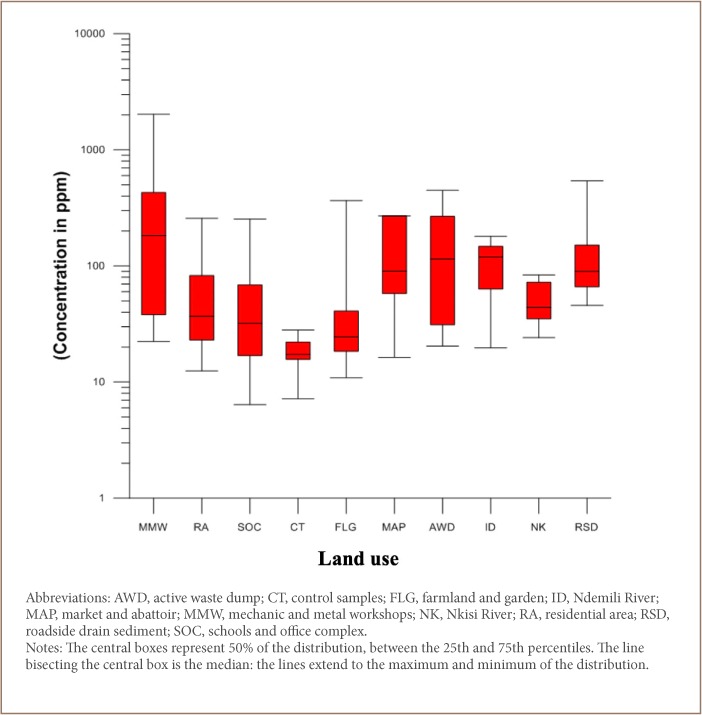
Total concentrations (ppm) of lead across different land use areas

The enrichment of Pb in the soils has been influenced by anthropogenic activities around the mechanic and metal workshops, as indicated by the higher concentrations of Pb in soils collected within the vicinity of these workshops compared to samples from other areas (*Table 3 and Figure 4*).

The concentration of Pb in the sediments also showed varying elevated concentrations across locations. The concentration of the roadside drain sediment ranged from 45.7–540.1 ppm, compared to the control (7.2–20.1 ppm), which showed a multiple-fold increase in the Pb concentration *(Table 3)*.

### Lead isotope geochemistry of lead in soils and sediments

The results of the ratios of lead isotope analyzed from the soils and sediments in the study area as well as that of coal, lead ore (galena), vehicle exhaust soot, and cell battery samples are presented in Table 6. The ratios are ^206^Pb/^204^Pb, ^207^Pb/^204^Pb, ^208^Pb/^204^Pb and ^206^Pb/^207^Pb. The results showed varying compositions in mean ratios across land use areas. The results of ^206^Pb/^204^Pb ratio were similar across all land use categories. The highest concentration ratio for ^206^Pb/^204^Pb was recorded in the lead ore (galena) (20.03). The mean ^206^Pb/^204^Pb ratio for residential area samples was 17.63, 17.56 for the school and office complex samples, 17.40 for the active waste dump samples, and 17.56 for the market and abattoir samples. The ratio for the mechanic and metal workshops (16.73) was low compared to the other land use areas. The ^206^Pb/^204^Pb isotope signature was similar to that of most land use types, except for Pb ore and vehicle exhaust. This implies that the Pb in these soils and sediments may have been enriched by a combination of more than one anthropogenic effect, hence the close resemblance with battery cell, coal and vehicle exhaust rations, and dissimilarity with the galena ore isotope ratio.

**Table 6:** Lead Isotope Compositions of Study Area Samples (N = 34) Compared to Selected Studies Globally

**Sample**	**^206^Pb/^204^Pb**	**^207^Pb/^204^Pb**	**^208^Pb/^204^Pb**
Residential area	17.63	15.25	35.70
Schools and office complex	17.56	15.18	36.40
Market and abattoir	17.56	15.32	36.09
Mechanic and metal workshops	16.73	14.58	33.93
Farmland and garden	18.46	15.85	38.03
Active waste dump	17.40	15.09	35.30
Ndemili River	17.42	15.15	35.24
Nkisi River	18.07	15.62	36.44
Roadside drain sediment	17.58	15.38	35.82
Control samples	17.82	15.54	37.59
Coal	17.64	14.41	34.83
Galena	20.03	16.32	39.22
Vehicle exhaust	18.09	15.21	36.03
Battery cell	17.23	14.43	34.15
Lake sediments, Hongfeng, Guizhou, China, N = 35^38^	19.22	15.72	38.95
Panagyurishte ore district, Bulgaria, N = 5^39^	18.35	15.53	38.45
Rhodope metallogenic zone, Bulgaria, N = 3^40^,^41^	18.31	15.65	38.55
Sediments from Xiangjiang River, Hunan Province, China, N = 23^42^	18.57	15.69	38.78
Sample from automobile exhaust, Hunan Province, China, N = 5^43^	18.09	15.58	37.74
Galena sample from Pb-Zn ore deposit district, Southern Hunan, China, N = 2^42^,^44^,^45^	18.51	15.67	38.83

**Figure 5:**
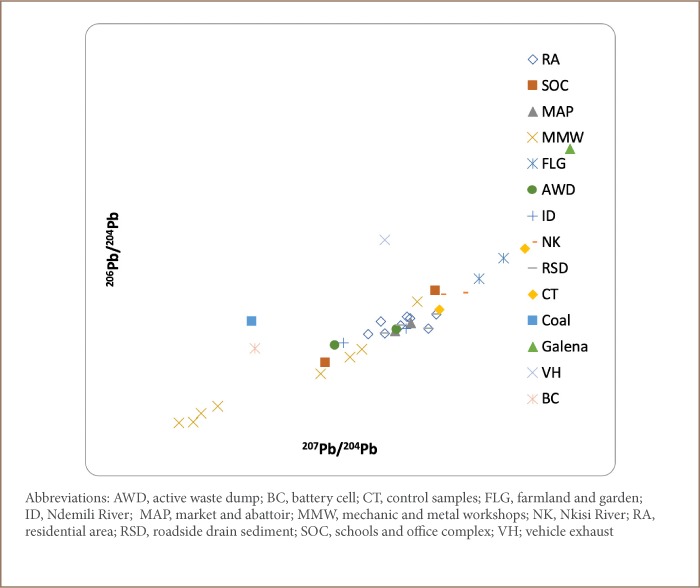
^206^Pb/^204^Pb vs ^207^Pb/^204^Pb correlation relationship for the sample media

**Figure 6:**
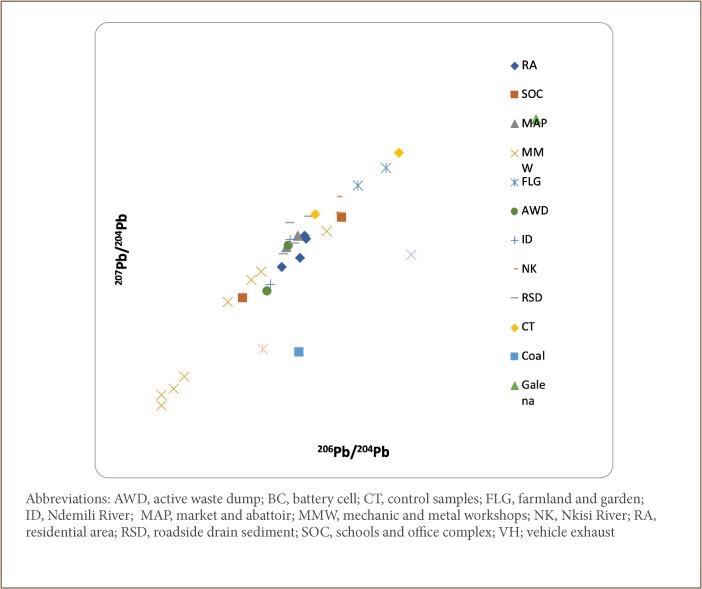
^207^Pb/^204^Pb vs ^206^Pb/^204^Pb correlation relationship for the sample media

A plot of the obtained ratios, ^206^Pb/^204^Pb vs ^207^Pb/^204^; ^207^Pb/^204^Pb vs ^206^Pb/^204^Pb; and ^208^Pb/^207^Pb vs ^208^Pb/^206^Pb *(Figures 5, 6 and 8)*, revealed clustering along two ends with samples from the residential area, schools and office complex, active waste dump, market and abattoir, and mechanic and metal workshops on one end, while galena, coal, vehicle exhaust and battery cell ratio signatures fell on the other end. The clustering of environmental Pb is an indication of the concentration of anthropogenic Pb in the samples compared to the reference materials.

**Figure 7:**
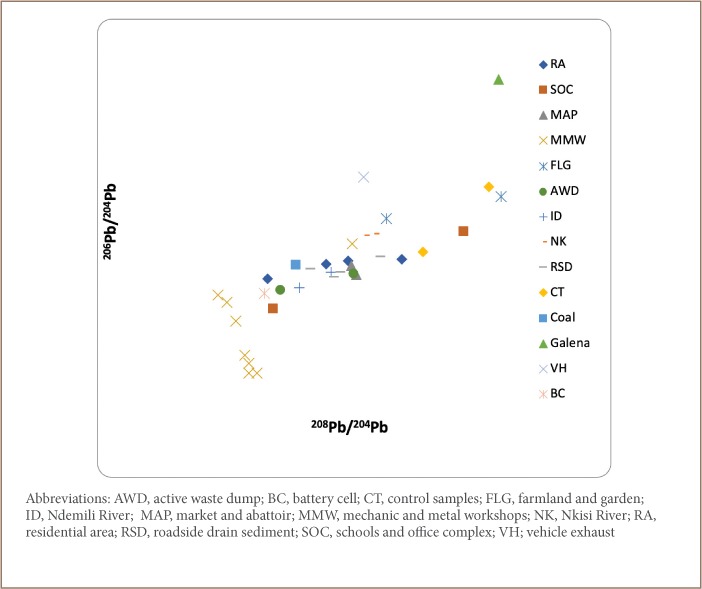
^206^Pb/^204^Pb vs ^208^Pb/^204^Pb correlation relationship for the sample media

**Figure 8:**
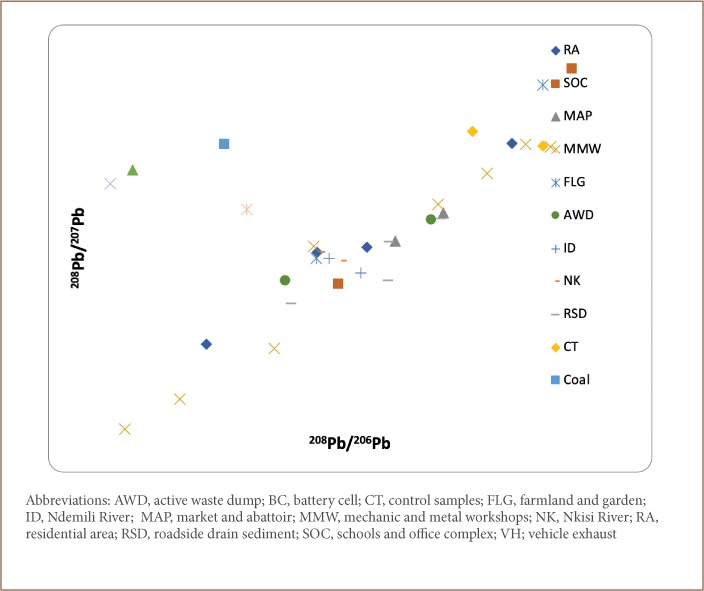
^208^Pb/^207^Pb vs ^208^Pb/^206^Pb correlation relationship for the sample media

### Fingerprinting sources of Pb in the environmental media

Table 6 shows the ratios of the various Pb isotopes (^206^Pb/^204^Pb, ^207^Pb/^204^Pb, ^208^Pb/^204^Pb and ^206^Pb/^207^Pb) evaluated. The ratios of ^206^Pb/^204^Pb were similar for all land use types (Table 6). The highest ratio of ^206^Pb/^204^Pb was obtained for galena (20.03), compared to the mean ratios for the residential area (17.63); schools and office complex (17.56); active waste dump (17.40) and market and abattoir (17.56). This implies that the Pb in the residential area, schools and office complex, active waste dump, and market and abattoir had anthropogenic rather than geogenic sources, which is the case for the galena ore.

Furthermore, the ^206^Pb/^204^Pb ratio for the mechanic and metal workshops (16.73) samples was found to be lower than those from the residential area, schools and office complex, active waste dump, market and abattoir, and for galena, an indication that the samples from the mechanic and metal workshops had greater anthropogenic-sourced ^204^Pb than the other land uses, indicating that this area is more greatly impacted than the other soil types.

In addition, the ^206^Pb/^204^Pb ratios for vehicle exhaust (18.09) and battery cells (17.23) were similar to those obtained for samples from the residential area, schools and office complex, active waste dump, and market and abattoir, an indication that the Pb in these samples had been contributed principally from wastes resulting from vehicle exhaust and battery cells.

The ^207^Pb/^204^Pb ratios for the residential area, schools and office complex, active waste dump, market and abattoir, and mechanic and metal workshops are similar to those of exhaust vehicle and different from those of galena and coal. This is further evidence that the Pb in the environmental samples have been significantly contributed by vehicular emissions. This is further corroborated by the ^206^Pb/^207^Pb ratios as shown in Table 6.

A comparison of the mean ratios of ^206^Pb/^204^Pb, ^207^Pb/^204^Pb, ^208^Pb/^204^Pb and ^206^Pb/^207^Pb from the present study was carried out with those of previous studies, as shown Table 6.^38–44^ From the results, it was observed that lake sediment from Hongfeng, Guizhou, China had a ^206^Pb/^204^Pb ratio (19.22) higher than that of the samples in the present study (soils: residential area (17.63) and farmland and garden (18.46) and sediments: Ndemili River (17.42) and Nkisi River (18.07)).^39^ The results in the present study were similar to the results from the Panagyurishte ore district (18.35), Rhodope Metallogenic zone (18.31), Xiangjiang River (18.57) and the galena sample from Pb-Zn ore deposits district in Southern Hunan (18.57).^40^,^41^,^42^,^43^,^44^,^45^^,46^

The ratios for vehicle exhaust in the present study (18.09) were the same for that of automobile exhaust in Hunan Province (18.09).^45^ There was also variation in the mean of ratios of ^207^Pb/^204^Pb, ^208^Pb/^204^Pb and ^206^Pb/^207^Pb as exhibited by the soils and sediments samples and other references in Table 5. This indicates that the enrichment of Pb in the geo-media may have resulted from multiple sources, but are mainly anthropogenic rather geogenic in origin.

## Conclusions

The soils and sediments in the Onitsha metropolis were studied for total Pb concentration and Pb isotopes contents. The soils and sediments are considered to be polluted relative to the background Pb concentration in the area, reference standards and governmental guidelines.

The soils were found to be more greatly enriched in anthropogenic-sourced Pb compared to sediments. With the relatively low pH established for garden and farmland soils, mobility of metals may be a problem, as they will be enhanced in such soils, making Pb potentially available for bioaccumulation in plants and crops. The higher Pb contents of the topsoil makes contact between humans and contaminated soils more likely. Topsoil could be blown as dusts particles that could easily be taken up via inhalation by humans and livestock.

It was also observed that several land use patterns in the city influenced the level of Pb pollution in the environmental media. The order of pollution of the soils from the various land use patterns is as follows: mechanic and metal workshops > active waste dump > market and abattoir > residential area > schools and office complex. The level of Pb in soils within schools and residential areas is particularly concerning, as these are locations where infants and children have regular contact with soils. This may eventually prove fatal for children and infants, as their low body mass may not be able to tolerate Pb toxicity over time. It is therefore crucial that immediate steps are taken to avert the potential danger posed by Pb in this area.

The inference from the study of Pb isotopes indicates that most of the Pb in the environmental media is anthropogenic ^204^Pb. This is significant as is indicates that Pb in the environmental media is predominantly contributed by non-sustainable environmental practices, such as indiscriminate waste dumps, hydrocarbon-based products emissions, by-products from mechanical workshops that have been haphazardly constructed in the city, and industrial plants located within urban areas.
